# Research progress and perspectives on the application of tyramide signal amplification-based multiplex immunohistochemistry/immunofluorescence: a bibliometrics analysis

**DOI:** 10.3389/fonc.2024.1473414

**Published:** 2025-01-24

**Authors:** Xiaotong Yu, Chen Huang, Yan Song, Chun Zhang, Debo You, XuRan Dong, DeFu Wu, Alan Keith Meeker, Hao Feng, Yuqing Wang

**Affiliations:** ^1^ Cancer Center of Peking University Third Hospital, Beijing, China; ^2^ Center of Basic Medical Research, Institute of Medical Innovation and Research, Peking University Third Hospital, Beijing, China; ^3^ Department of Ophthalmology, Peking University Third Hospital, Beijing, China; ^4^ Beijing Key Laboratory of Restoration of Damaged Ocular Nerve, Peking University Third Hospital, Beijing, China; ^5^ Oncology Tissue and Imaging Services, Johns Hopkins University Sidney Kimmel Comprehensive Cancer Center, Baltimore, MD, United States

**Keywords:** multiplex immunohistochemistry/immunofluorescence, tyramide signal amplification, bibliometric analysis, CiteSpace, VOSviewer

## Abstract

**Background and aims:**

Multiplex immunohistochemistry/immunofluorescence (mIHC/IF), which uses the tyramide signal amplification (TSA) technique, enables sequential staining of multiple targets in formalin-fixed paraffin-embedded (FFPE) samples without worrying about cross-reactivity. This approach has received considerable attention from researchers over the past decades. This article aims to provide a bibliometric analysis of the research progress and perspectives on the application of TSA-based mIHC/IF.

**Methods:**

We collected all the TSA-based mIHC/IF documents published between 2007 and 2023 from the Web of Science Core Collection (WoSCC) database. CiteSpace, VOSviewer and Bibliometrix R Package were used to perform the bibliometrics analysis, including details about annual publications, countries, institutions, authors, journals, and research topics and hotspots.

**Results:**

A total of 873 relevant publications (811 articles and 62 reviews) with a time span of 17 years (2007-2023) were obtained. The number of annual publications started to increase rapidly since 2016. The United States (307, 35.17%) and the People’s Republic of China (297, 34.02%) are the top two listed countries for both the number of articles produced and the citations. The University of Texas System (53, 6.07%) was the most productive institution. Integrating these results of hotspot and frontier analysis, TSA-based mIHC/IF provides significant benefits, particularly in neurology, cancer and immunology.

**Conclusion:**

This study conducted a comprehensive bibliometric analysis for the use of TSA-based mIHC/IF. As TSA-based mIHC/IF and its associated imaging systems and analytic software progress, it will become the most promising tool for describing the variety of the whole tissue for a better understanding of pathological or physiological behavior.

## 1 Introduction

Multiplex immunohistochemistry/immunofluorescence (mIHC/IF) technologies are a suite of technologies for the simultaneous visualization of multiple biomarkers in a single tissue section (1) and can be divided into five classes: stain-removal technologies, fluorophore inactivation technologies, multiplex signal amplification, DNA barcoding technologies, and mass cytometry (2). Despite being created more than 20 years ago, tyramide signal amplification (TSA)-based mIHC/IF is still commonly utilized, identifying up to eight markers in a single formalin-fixed paraffin-embedded (FFPE) section using a cyclic staining protocol with tyramide-conjugated fluorophores (3–5). The extremely sensitive enzyme-catalyzed procedure detects modest levels of expression by increasing the signal above background tissue autofluorescence. Iterative cycles of antibody staining and antibody stripping by heat-induced epitope retrieval, generally conducted in the microwave, allow the use of primary antibodies from the same species with no species cross-reactivity (3, 6). Furthermore, TSA-based mIHC/IF is significantly photostable compared to conventional immunofluorescence, allowing slides to be stored and rescanned one year after staining without significant loss of signal (7). Nowadays, the procedure of TSA-based mIHC/IF, image acquisition, spectral unmixing, and data analysis have been optimized, making it suitable for both manual and automated assays (3, 6, 8, 9), and the reproducibility and validation of TSA-based mIHC/IF against traditional IHC have been verified (10–13). TSA-based mIHC/IF detects the co-expression of several molecules on single cells, assesses the distribution, abundance, and heterogeneity of expression of various cell types in tissues, and identifies spatial correlations between them. Several scholars have reviewed the application of mIHC/IF approach in research areas such as immune microenvironment and tumor immunotherapy (2, 14–16), suggesting that TSA-based mIHC/IF can increase researchers’ understanding of physiological processes and diseases at the molecular and cellular levels, making them crucial for disease categorization, etiology, diagnosis, and therapy. However, there is currently little information available on the applications, demographic distribution and general development trend of this innovative technology.

One of the most significant responsibilities in improving a field is to draw conclusions from prior research. Bibliometrics is a well-established bioinformatics quantitative tool that rigorously applies statistical and mathematical methods to large amounts of unstructured data, using the global document characteristics and literature landscape as the object of study, to decipher and map the cumulative scientific knowledge and evolution of a defined field (17). Several software integrating computer engineering, big data applications and statistics have been widely applied in this field, including CiteSpace, VOSviewer and Bibliometrix R Package (18). To date, no bibliometric analyses have been conducted in the TSA-based mIHC/IF field. In this particular study, we have collected literature that applied or reviewed TSA-based mIHC/IF from the Web of Science Core Collection (WoSCC) database and performed a systematic bibliometric analysis of this method, and attempted to provide an overview and a different perspective to help understand the history of scientific activities and the evolution of hotspots, identify current research interests, guide potential future research directions and emerging trends, and promote further generalization of its application and development.

## 2 Materials and methods

### 2.1 Data source and search strategy

**Figure 1:**
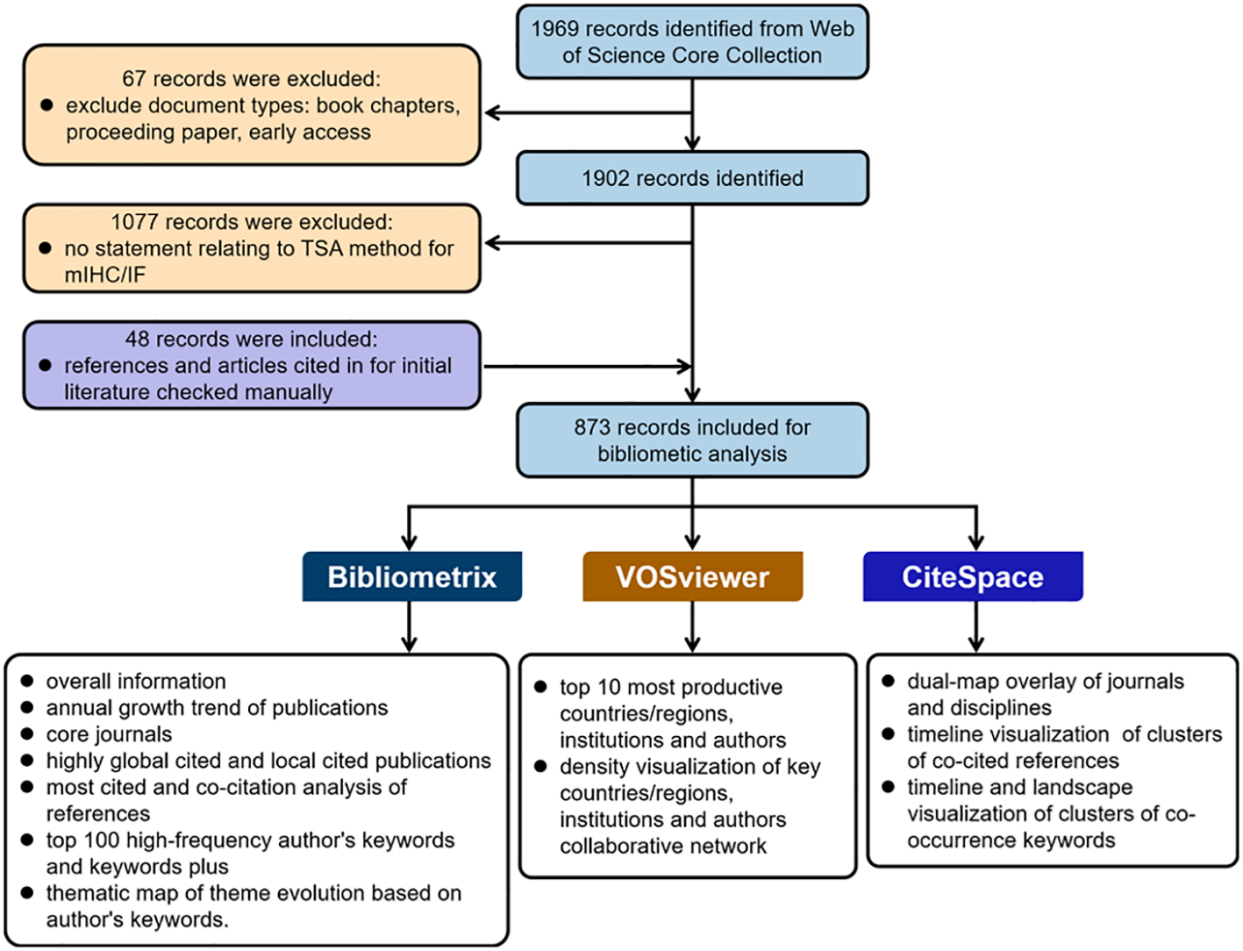
Flowchart of the data preparation and bibliometric analysis process.

The literature content used in this study was extracted from the WoSCC databases including SSCI, SCI-Expanded, CPCI-S, A&HCl, ESCI, CPCI-SSH, CCR-Expanded and IC. The search strategy was: TS=(“multiplex* immunofluorescence” OR “multiplex* immunohistochemistry” OR “multiplex* immunohistochemistry/immunofluorescence” OR “multiplex* immunohistochemistry/multiplex* immunofluorescence” OR “multiplex* fluorescent immunohistochemistry” OR “multiplex* IHC” OR “multiplex* IF” OR “multispectral fluorescent immunohistochemistry” OR “multiplex* immunohistochemical”) OR TS=(tyramide-based OR TSA-based OR “tyramide signal amplification”). Only “Article” and “Review Article” were included in the analysis, and the language of the literature was set to English. The publication period was from the start of the database until 21 December 2023. The “Full Record and Cited References” of the original data were also extracted in “Plain Text” format and downloaded on 21 December 2023. In addition, the full text of these records was examined by two authors to determine whether it includes any application or discussion of TSA-based mIHC/IF in the literature. Meanwhile, the references and articles cited in the original literature were manually checked to avoid missing articles. Any discrepancies were resolved by discussion. The flowchart of the data preparation and bibliometric analysis process is shown in **Figure 1**.

### 2.2 Bibliometric analysis

The data were imported into the Bibliometrix (4.1) R package (19), VOSviewer (version 1.6.16) (20) and CiteSpace (version 6.1.R3) (21, 22) for bibliometric analysis and graphical visualization.

The Bibliometrix (4.1) R package (19) performs a complete set of literature information analysis and the visualization of results. In this study, the Bibliometrix (4.1) R package was used to obtain the data of overall information, annual growth trend of publications, core journals, highly global cited and local cited publications, most cited and co-citation analysis of references, top 100 high-frequency author’s keywords and keywords plus, and thematic map of theme evolution based on author’s keywords. The thematic map (19) is a two-dimensional strategy diagram that divides the development of research themes into four quadrants according to their centrality (plotted on the x-axis) and density (plotted on the y-axis). The algorithm of the thematic map uses co-word and h-index indicators, then creates networks of keywords and calculates the relationship among them. Centrality measures the degree of interaction that a keyword network has with other keyword networks. Density measures the internal strength of a network, implying the closeness of these words to each other. Subsequently, the keywords were grouped according to subject areas and the subjects were distributed according to centrality and density.

VOSviewer (20) is a software tool for constructing and visualizing bibliometric co-occurrence networks based on citation, bibliographic coupling, co-citation, or co-authorship relations. It was used to obtain the data of the top 10 most productive countries/regions, institutions and authors, and to perform co-occurrence analysis of countries/regions, institutions and authors in density visualization.

CiteSpace (21, 22) is a Java application for visualizing and analyzing trends and patterns in scientific literature, with a focus on finding critical points in the development of a field or domain, especially intellectual inflection points and pivots. It provides structural and temporal analyses of a variety of networks derived from scientific publications to facilitate the understanding and interpretation of network patterns and historical patterns, including dual-map overlay of journals and disciplines, visualization networks and clusters of co-cited references and co-occurrence keywords.

## 3 Results

### 3.1 Global overview of publications

According to the inclusion and exclusion criteria, this study comprised 873 publications (811 articles and 62 reviews) from the WOSCC database during a 17 years period (2007-2023), with an annual growth rate of 39.3%. The total number of citations in the retrieved publications totaled 13993, with an average of 20.61 citations per publication. In addition, 29 papers were cited more than 100 times. In total, the retrieved material was published in 296 journals globally by approximately 7245 authors representing 1401 institutions in 52 countries/regions.

### 3.2 Annual growth trend of publications

**Figure 2:**
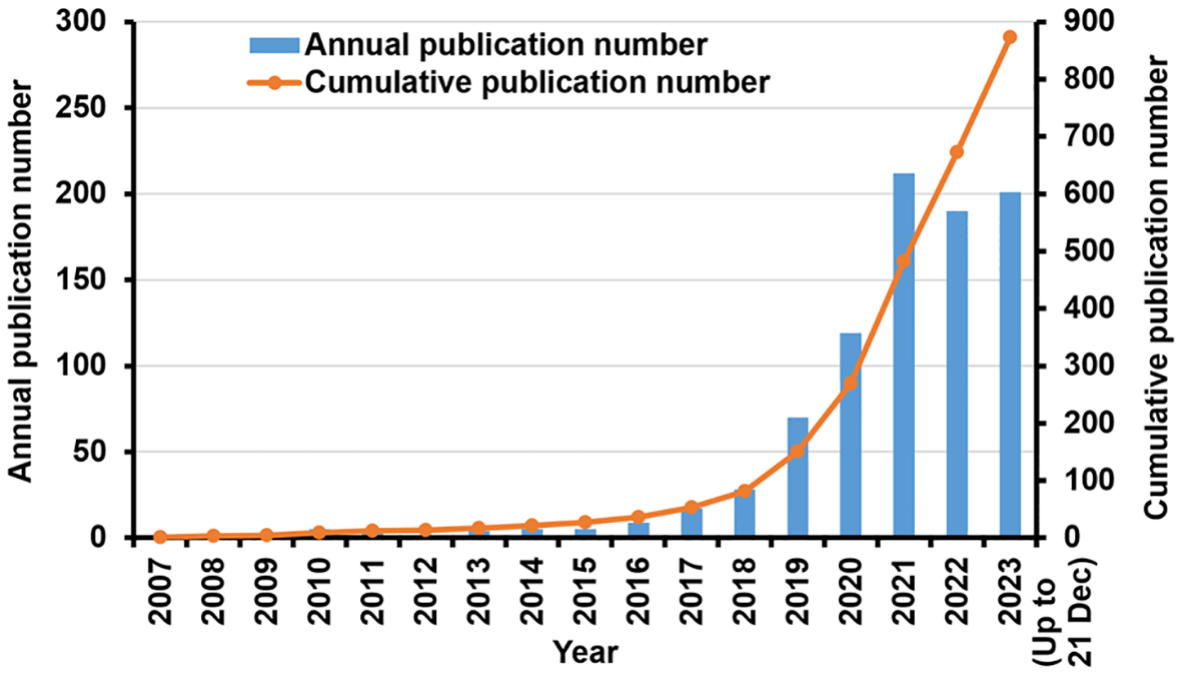
Research in TSA-based mIHC/IF method: Annual scientific production and cumulative number of publications.

As shown in **Figure 2**, the overall trend of research on the use of TSA-based mIHC/IF has been expanding. The first article appeared in the *Journal of Histochemistry & Cytochemistry* in 2007 (23). Toth ZE and Mezey E were the first to demonstrate the simultaneous visualization of multiple antigens with TSA using antibodies from the same species (23). Between 2007 and 2015, there were just a few publications. Since 2016, the number of annual publications has increased substantially. Between 2017 and 2023, 846 publications were released, accounting for 96.91% of all contained materials. The number of yearly publications grew progressively, from 119 in 2020 to 212 in 2021. As of December 21, 201 publications have been published in 2023.

### 3.3 Contributions of countries/regions, institutions and authors to global publications

**Figure 3:**
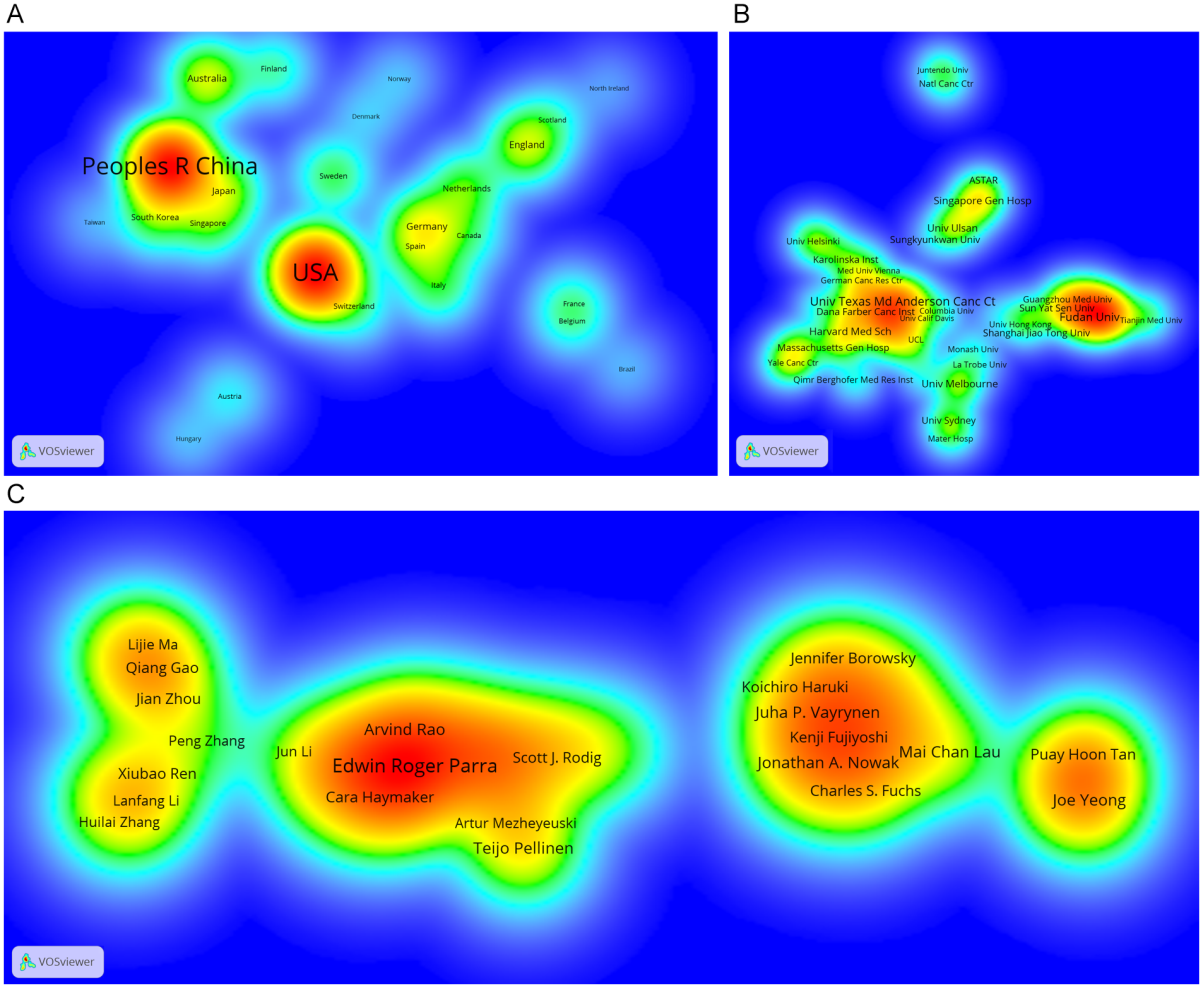
Density visualization of the most important countries/regions **(A)**, institutions **(B)** and authors **(C)** in the co-occurrence network generated by VOSviewer. Only those countries/regions, institutions and authors with at least 5 records are shown in the network.

Four European countries, four Asian countries, one North American country, and one Oceania country make up the top ten most productive nations (**Table 1**). The two most productive countries, the United States (307 publications, 35.17%) and the People’s Republic of China (297 publications, 34.02%), contributed a total of 573 publications (65.64%). **Table 2** shows the top 10 most productive institutions, with two colleges tied for tenth place. Similarly, the vast majority of the 11 institutions are from the United states (5 institutions) and the People’s Republic of China (4 institutions). Seven of the top nine writers with the most publications, seven are from the United States, three from Singapore and one from South Korea, with output years beginning in 2017 or later (**Table 3**). Parra Edwin Roger from the University of Texas MD Anderson Cancer Center published the most papers (28 publications). As shown in **Figure 3**, the clusters for key countries/regions, institutions, and authors are shown in density visualizations generated by VOSviewer.

**Table 1:** Top 10 productive countries with the most publications applying TSA-based mIHC/IF.

Rank	Country	Publications	Production year start
1	USA	307	2007
2	Peoples R China	297	2017
3	Australia	61	2017
4	Germany	58	2008
5	England	56	2010
6	Japan	47	2017
7	Netherlands	38	2014
8	South Korea	35	2015
9	Italy	32	2013
10	Sweden	32	2013

**Table 2:** Top 10 productive institutions with the most publications applying TSA-based mIHC/IF.

Rank	Institution	Country	Publications	Production year start
1	University of Texas MD Anderson Cancer Center	USA	53	2015
2	Fudan University	Peoples R China	39	2019
3	Sun Yat Sen University	Peoples R China	36	2018
4	Harvard Medical School	USA	28	2016
5	Dana Farber Cancer Institute	USA	27	2017
6	Brigham Women S Hospital	USA	26	2017
7	Capital Medical University	Peoples R China	26	2018
8	Memorial Sloan Kettering Cancer Center	USA	26	2015
9	Shanghai Jiao Tong University	Peoples R China	25	2017
10	Singapore General Hospital	Singapore	24	2018
10	University of Ulsan	South Korea	24	2017

**Table 3:** Top 9 productive authors with the most publications applying TSA-based mIHC/IF.

Rank	Author	Institution	Country	Publications	Production year start
1	Edwin Roger Parra	University of Texas MD Anderson Cancer Center	USA	28	2017
2	Sang-Yeob Kim	University of Ulsan	South Korea	19	2019
3	Ignacio I. Wistuba	University of Texas MD Anderson Cancer Center	USA	18	2017
4	Mai Chan Lau	ASTAR Institute of Molecular and Cell Biology	Singapore	14	2020
4	Joe Yeong	Singapore General Hospital	Singapore	14	2018
4	Jeffrey Chun Tatt Lim	ASTAR Institute of Molecular and Cell Biology	Singapore	14	2018
7	Arvind Rao	University of Michigan	USA	12	2017
7	David l. Rimm	Yale University	USA	12	2019
9	Jonathan A. Nowak	Harvard Medical School	USA	11	2019
9	Mei Jiang	University of Texas MD Anderson Cancer Center	USA	11	2017
9	Janis M. Taube	Johns Hopkins University	USA	11	2018

### 3.4 Core journals and disciplines

In all, 873 retrieved articles were published in 294 journals. **Table 4** lists the top 10 journals that published the most literature, accounting for 38.37% of the total. *Journal for Immunotherapy of Cancer* was the most active journal in this topic, followed by *Frontiers in Immunology* and *Cancers*. All 11 journals have a Journal Impact Factor 2022 (JIF2022) >3.00, with *Nature Communications* having the highest IF of 16.6. According to the JIF Quartile 2022 standards, the *Journal for Immunotherapy of Cancer*, *Cancers*, *Clinical Cancer Research*, *Oncoimmunology* and *British Journal of Cancer* are classified as Q1 in the JCR category, which are professional and active journals in the field of oncology. In addition, a few articles were published in journals with a high JIF of more than 40, such as *Science* (JIF2022 = 56.9), *Lancet Oncology* (JIF2022 = 51.1), *Cancer Cell* (IF2022 = 50.3) and *Nature Methods* (JIF2022 = 8).

**Table 4:** Top 10 core journals with the most publications applying TSA-based mIHC/IF.

Rank	Journal	Publications	JIF (2022)	Category	JIF quartile (2022)	Production year start
1	Journal for Immunotherapy of Cancer	67	10.9	Immunology/Oncology	Q1/Q1	2015
2	Frontiers in Immunology	56	7.3	Immunology	Q1	2020
3	Cancers	45	5.2	Oncology	Q2	2019
4	Clinical Cancer Research	38	11.5	Oncology	Q1	2017
5	Frontiers in Oncology	33	4.7	Oncology	Q2	2019
6	International Journal of Molecular Sciences	22	5.6	Biochemistry & molecular biology/ Chemistry, multidisciplinary	Q1/Q2	2015
7	Oncoimmunology	21	7.2	Immunology/Oncology	Q1/Q1	2017
8	Scientific Reports	19	4.6	Multidisciplinary sciences	Q2	2015
9	Journal of Translational Medicine	12	7.4	Medicine, Research & Experimental	Q1	2017
10	British Journal of Cancer	11	8.8	Oncology	Q1	2016
10	Nature Communications	11	16.6	Multidisciplinary sciences	Q1	2017

**Figure 4:**
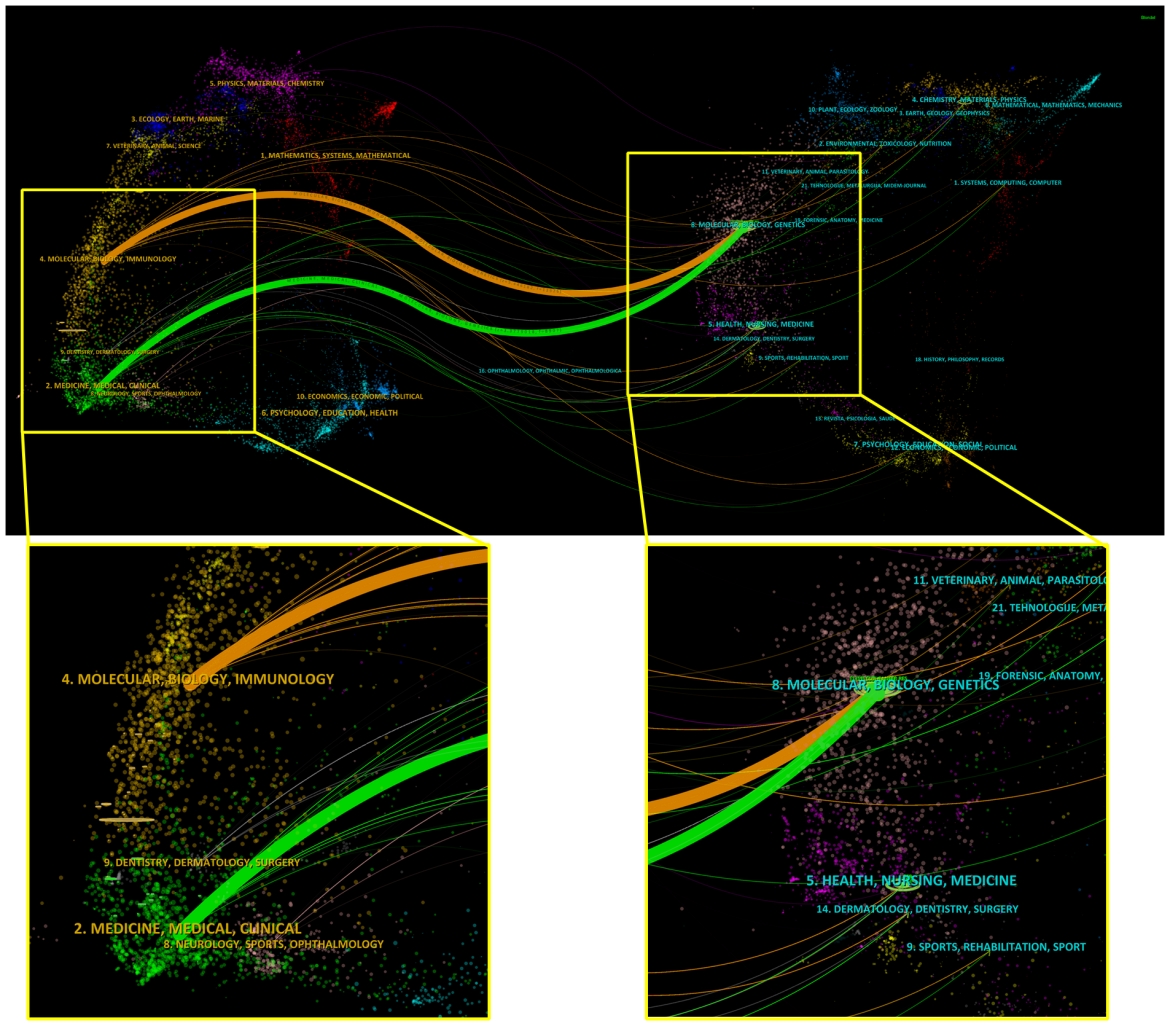
Dual map overlay and corresponding disciplines of citing and cited publications generated by CiteSpace. Labels indicate corresponding disciplines of citing and cited publications. The citing and cited trajectory using the z-score function.

Each journal included in the WoSCC databases is classified into at least one of the subject categories related to a certain research field. TSA-based mIHC/IF was applied to 65 specialties, depending on the journal field. Among them, Oncology accounts for the largest number of publications (417 publications), followed by Immunology (200 publications), Pathology (56 publications), Multidisciplinary Sciences (56 publications) and Medicine Research Experimental (55 publications). In addition, a dual-map overlay of journals is created to illustrate the citation link between journals and disciplines by marking the journal’s topic area (**Figure 4**). The present map shows two major citation categories, suggesting that majority of articles were published in the domains of ‘Molecular, Biology, Immunology’ and ‘Medicine, Medical, Clinical’, and that they mainly cited journals in the fields of ‘Molecular, Biology, Genetics’.

### 3.5 Research topics and hotspots

#### 3.5.1 Highly total global cited and local cited publications

The most referenced papers, whether worldwide or locally, have acted as watershed moments for key discoveries and research boosters.

The total global citations (TC) and the average number of citations per year (Nc/year) may reflect the relevance and interest of other researchers in a certain study topic. The top 20 most cited publications (13 articles and 7 reviews) are ranked in **Table 5** according to the total number of citations in the WoSCC databases. Eight publications had the Nc/year values greater than 50, seven of which were on oncology research topics and the remainder were COVID-19.

**Table 5:** Top 20 publications with the highest number of total citations applying TSA-based mIHC/IF.

Rank	Title	First author	Journal	Year	Type	Total Global Citations	Nc/year
1	Predictive biomarkers for checkpoint inhibitor-based immunotherapy	Gibney GT	*Lancet Oncol*	2016	Review	1108	138.5
2	Multiplexed immunohistochemistry, imaging, and quantitation: A review, with an assessment of Tyramide signal amplification, multispectral imaging and multiplex analysis	Stack EC	*Methods*	2014	Review	478	47.8
3	Spatial computation of intratumoral T cells correlates with survival of patients with pancreatic cancer	Carstens JL	*Nat Commun*	2017	Article	356	50.86
4	Comparison of biomarker modalities for predicting response to PD-1/PD-L1 checkpoint blockade: a systematic review and meta-analysis	Lu S	*JAMA Oncol*	2019	Review	343	68.6
5	Redefining tumor-associated macrophage subpopulations and functions in the tumor microenvironment	Wu KY	*Front Immunol*	2020	Review	259	64.75
6	CD103+ tumor-resident CD8+ T cells are associated with improved survival in immunotherapy-naïve melanoma patients and expand significantly during anti-PD-1 treatment	Edwards J	*Clin Cancer Res*	2018	Article	254	42.33
7	Overview of multiplex immunohistochemistry/immunofluorescence techniques in the era of cancer immunotherapy	Tan WCC	*Cancer Commun*	2020	Review	234	58.5
8	Topological analysis reveals a PD-L1-associated microenvironmental niche for Reed-Sternberg cells in Hodgkin lymphoma	Carey CD	*Blood*	2017	Article	230	32.86
9	Single-cell transcriptomic architecture and intercellular crosstalk of human intrahepatic cholangiocarcinoma	Zhang M	*J Hepatol*	2020	Article	200	50
10	Tissue-specific immunopathology in fatal COVID-19	Dorward DA	*Am J Resp Crit Care*	2021	Article	176	58.67
11	PD1Hi CD8+ T cells correlate with exhausted signature and poor clinical outcome in hepatocellular carcinoma	Ma J	*J Immunother Cancer*	2019	Article	175	35
12	Validation of multiplex immunofluorescence panels using multispectral microscopy for immune-profiling of formalin-fixed and paraffin-embedded human tumor tissues	Parra ER	*Sci Rep*	2017	Article	174	24.86
13	Interleukin-17-induced neutrophil extracellular traps mediate resistance to checkpoint blockade in pancreatic cancer	Zhang Y	*J Exp Med*	2020	Article	171	42.75
14	Spatial omics and multiplexed imaging to explore cancer biology	Lewis SM	*Nat Methods*	2021	Review	167	55.67
15	Simultaneous visualization of multiple antigens with tyramide signal amplification using antibodies from the same species	Toth ZE	*J Histochem Cytochem*	2007	Article	155	9.12
16	Crucial role of CB2 cannabinoid receptor in the regulation of central immune responses during neuropathic pain	Racz I	*J Neurosci*	2008	Article	153	9.56
17	Eight-color multiplex immunohistochemistry for simultaneous detection of multiple immune checkpoint molecules within the tumor microenvironment	Gorris MAJ	*J Immunol*	2018	Article	141	23.5
18	T-cell localization, activation, and clonal expansion in human pancreatic ductal adenocarcinoma	Stromnes IM	*Cancer Immunol Res*	2017	Article	135	19.29
19	Mobilization of CD8+ T cells via CXCR4 blockade facilitates PD-1 checkpoint therapy in human pancreatic cancer	Seo YD	*Clin Cancer Res*	2019	Article	126	25.2
20	Tumor-infiltrating lymphocytes and their prognostic value in cutaneous melanoma	Maibach F	*Front Immunol*	2020	Review	124	31

**Figure 5:**
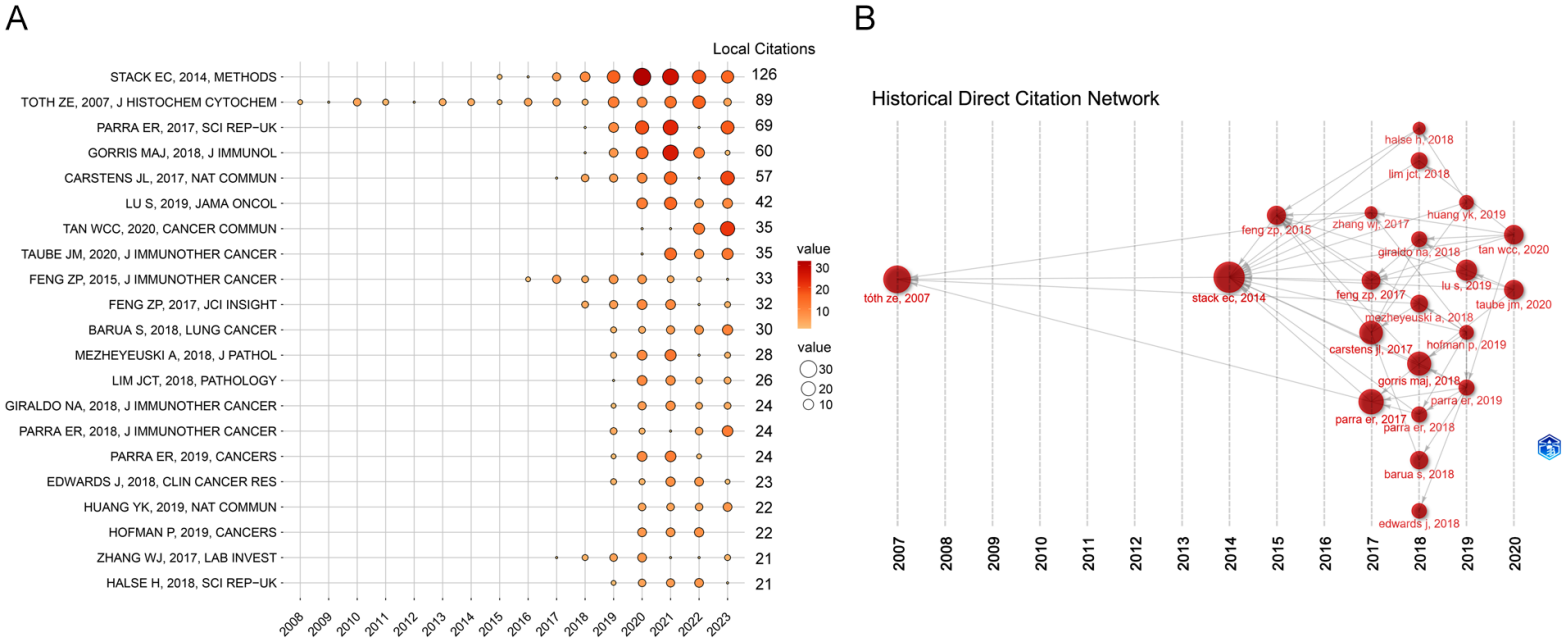
Annual citation **(A)** and historical direct citation network **(B)** of the top 20 publications in the Local Citation Score (LCS) ranking. **(A)** The size and color of the circles represent the annual LCS of the publication, with larger circles and redder colors indicating a higher LCS. **(B)** Historical direct citation network generated by the Bibliometrix R package. Each node in the historical direct citation network represents a key publication, and the directional arrow indicates the citation association between the two publications. The size of each node is proportional to the frequency of that publication among the other 19 publications.

The Local Citation Score (LCS) is an essential indicator of a publication’s influence in this particular research field. **Figure 5** depicts the details of the annual citation and the historical direct citation network of the top 20 publications in the LCS ranking. The majority of the high LCS publications have been published and referenced since 2017, indicating that TSA-based mIHC/IF has gained extensive attention and application among academics since then (**Figure 5A**). In the historical direct citation network, each node representing a cited paper is scaled according to the LCS, and the linkages reflect citations between publications (**Figure 5B**). The review published by Stack EC et al. (3) and the article published by Toth ZE et al. (23) in 2007 received the two highest LCS ratings as the most important early literature, representing two of the earliest publications reporting and reviewing this technique. Two automated staining protocols have also attracted a great deal of attention from the research community (8, 24). Oncology, the main application area of TSA-based mIHC/IF, accounts for the remaining 17 publications.

#### 3.5.2 Most cited and co-citation analysis of references

**Figure 6:**
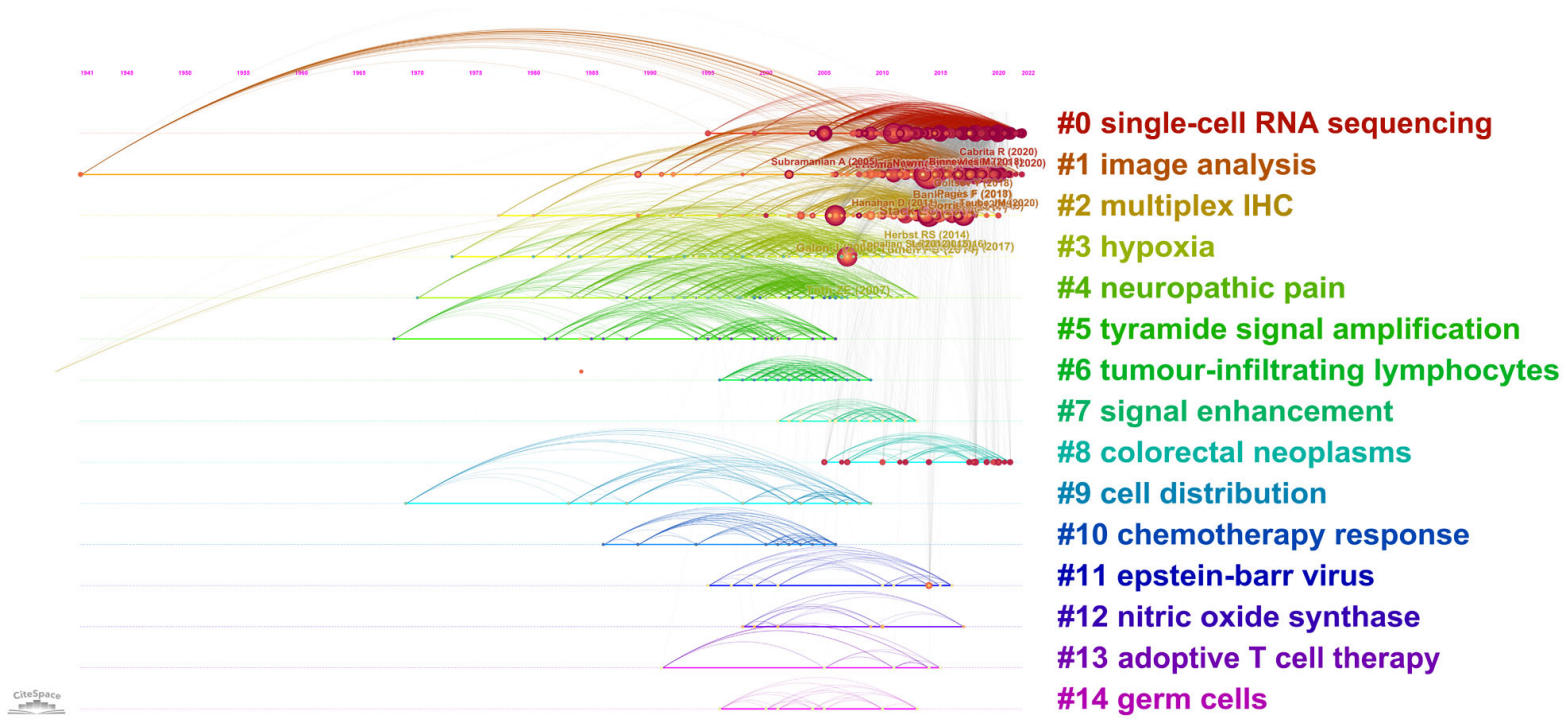
Timeline visualization of reference co-citation analysis generated by CiteSpace.

A total of 29173 references were involved in 873 publications. The top 10 most cited references are listed in **Table 6**. Six of them were published in very influential publications, including *CA: A Cancer Journal for Clinicians*, *Science*, *Nature*, *Nature Reviews Cancer*, and *Nature Communications*. In the visualization network of co-cited references, the nodes representing the references are grouped into 15 particular clusters with the greatest K-values (**Figure 6**). The timeline view illustrates the evolutionary path of these conspicuous groupings. Each circle represents a major reference in a certain cluster, whereas the citation tree rings of varying widths on the timeline show citation rates. Large nodes are frequently noted in each time slice. The number of cluster labels is inversely proportional to the number of publications inside in each cluster. Five clusters, including ‘#0 single-cell RNA sequencing’, ‘#1 image analysis’, ‘#2 multiplex IHC’, ‘#3 hypoxia’ and ‘#4 neuropathic pain’, are larger than 100, suggesting that they have received special attention.

**Table 6:** Top 10 most cited references for publications applying TSA-based mIHC/IF.

Rank	Citations	Title	First author	Journal	Publication year	Type	DOI
1	126	Multiplexed immunohistochemistry, imaging, and quantitation: a review, with an assessment of Tyramide signal amplification, multispectral imaging and multiplex analysis	Stack EC	Methods	2014	Review	10.1016/J.YMETH.2014.08.016
2	89	Simultaneous visualization of multiple antigens with tyramide signal amplification using antibodies from the same species	Toth ZE	J Histochem Cytochem	2007	Article	10.1369/JHC.6A7134.2007
3	82	PD-1 blockade induces responses by inhibiting adaptive immune resistance	Tumeh PC	Nature	2014	Article	10.1038/NATURE13954
4	69	Validation of multiplex immunofluorescence panels using multispectral microscopy for immune-profiling of formalin-fixed and paraffin-embedded human tumor tissues	Parra ER	Sci Rep-UK	2017	Article	10.1038/S41598-017-13942-8
5	63	Type, density, and location of immune cells within human colorectal tumors predict clinical outcome	Galon J	Science	2006	Article	10.1126/SCIENCE.1129139
6	60	Eight-Color Multiplex Immunohistochemistry for Simultaneous Detection of Multiple Immune Checkpoint Molecules within the Tumor Microenvironment	Gorris MAJ	J Immunol	2018	Article	10.4049/JIMMUNOL.1701262
7	57	Spatial computation of intratumoral T cells correlates with survival of patients with pancreatic cancer	Carstens JL	Nat Commun	2017	Article	10.1038/NCOMMS15095
8	56	Global cancer statistics	Jemal A	Ca-Cancer J Clin	2011	Article	10.3322/CAAC.20107
9	53	The immune contexture in human tumours: impact on clinical outcome	Fridman WH	Nat Rev Cancer	2012	Review	10.1038/NRC3245
10	50	QuPath: Open source software for digital pathology image analysis	Bankhead P	SCI REP-UK	2017	Article	10.1038/S41598-017-17204-5

#### 3.5.3 High-frequency, co-occurrence analysis and thematic map of keywords

**Figure 7:**
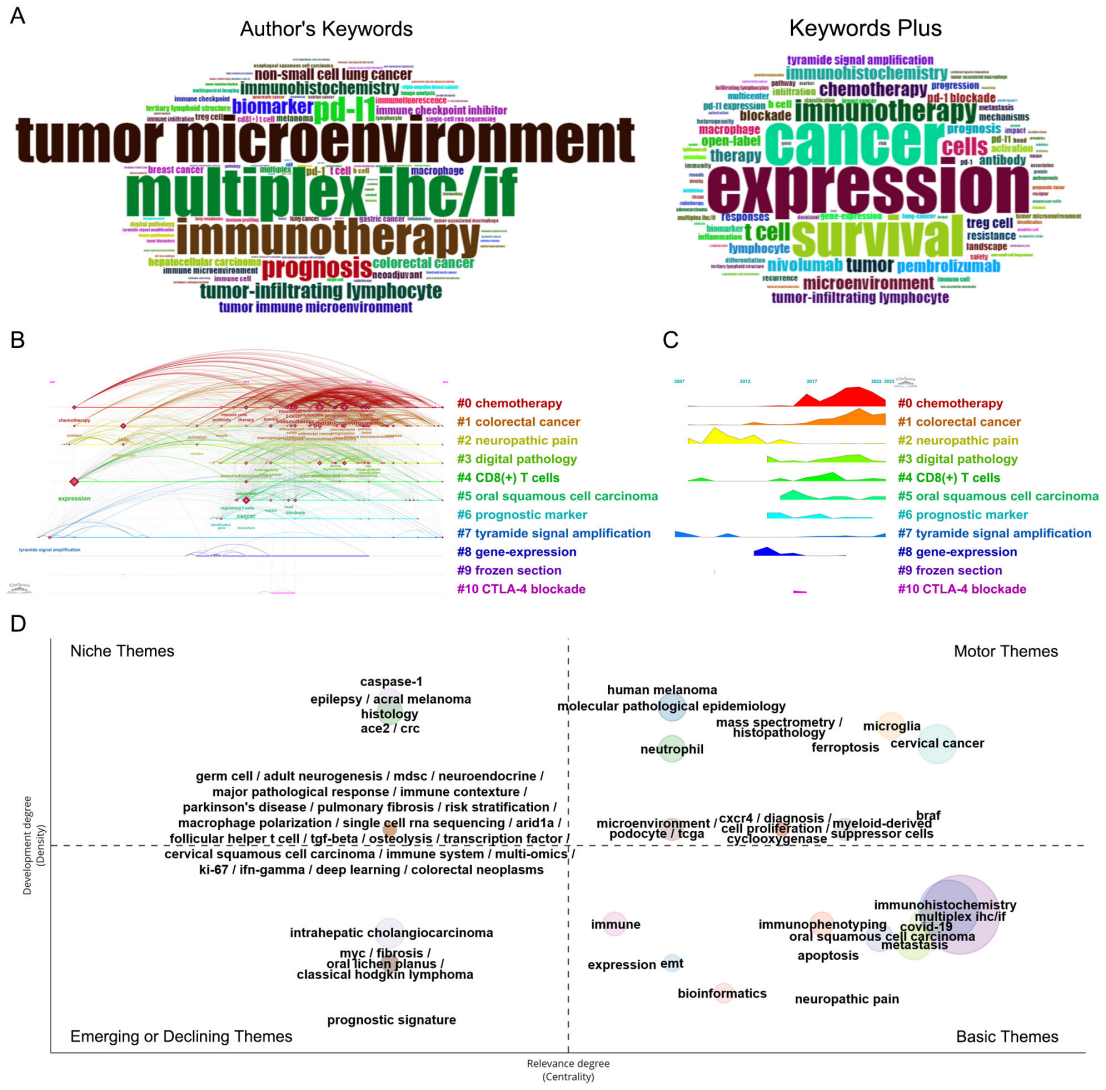
Co-occurring analysis of keywords of publications applying the TSA-based mIHC/IF method. **(A)** Word cloud of the top 100 high-frequency authors keywords and keywords plus generated by the Bibliometrix R package. **(B)** Timeline view of the significant clusters of keywords generated by CiteSpace. **(C)** Landscape of the significant clusters of keywords generated by CiteSpace. **(D)** Thematic map of keywords generated by the Bibliometrix R package.

Keywords are focused representations of a publication’s fundamental topic. A total of 1837 author keywords and 1906 additional keywords were collected from the 873 publications. After integrating synonyms, the word clouds in **Figure 7A** reflect the top 100 most often used author’s keywords and keywords plus, with font size positively correlated with frequency. In addition to multiplex IHC/IF, the following keywords appear with a frequency greater than 50: expression, cancer, survival, tumor microenvironment, immunotherapy, pd-l1, T cell, tumor, chemotherapy, nivolumab, microenvironment, immunohistochemistry, pembrolizumab, therapy.

Co-occurrence keywords can reveal meaningful knowledge components and insights based on the pattern and strength of links between keywords, indicating the hotspots and latest trends in the research field by uncovering knowledge mappings. After clustering the network map of keyword co-occurrence, 11 significant clusters with the highest K-value are generated with the hierarchical cluster labels of the most relevant terms (**Figures 7B, C**). The timeline view and landscape of the clusters demonstrate how TSA-based mIHC/IF have changed over time in various applications. More than 100 keywords are found in three clusters, “#0 chemotherapy”, “#1 colorectal cancer”, and “# neuropathic pain” The Cluster “#2 neuropathic pain” was common in former years. Chemotherapy and colorectal cancer remain typical applications for TSA-based mIHC/IF.

As shown in **Figure 7D**, the thematic map divides 65 clusters of author’s keywords into four quadrants according to their centrality and density rank: niche (top left, 30 clusters), motor (top right, 17 clusters), emerging or declining (left bottom, 6 clusters), and basic themes (right bottom, 12 clusters). The size of the bubble corresponds to the number of terms in the cluster. Each cluster is assigned with a descriptive label generated from high-frequency keywords that serve as primary themes and illustrate the breadth of application of TSA-based mIHC/IF. Partially overlapping clusters have the same centrality and relevance, with their labels separated by slashes. The basic themes in the bottom right quadrant are fundamental, significant, universal and cross-cutting to the research area. Among them, five clusters, including ‘immunohistochemistry’, ‘multiplex IHC/IF’, ‘covid-19’, ‘metastasis’ and ‘oral squamous cell carcinoma’ have the highest relevance according to the highest centrality. The motor themes, located in the top right quadrant, consist of 17 well-developed and essential clusters for organizing the study topic. The clusters labeled as ‘human melanoma’, ‘molecular pathological epidemiology’, ‘microglia’, ‘histopathology’ and ‘mass spectrometry’, have the highest density, while ‘cervical cancer’, ‘braf’, ‘microglia’, ‘ferroptosis’ and ‘myeloid-derived suppressor cells’ demonstrate the highest centrality. Niche themes in the upper left quadrant, characterized by low centrality and high density, are highly developed and isolated or specialized themes, such as ‘caspase-1’, ‘epilepsy’, ‘acral melanoma’, ‘ace2’ and ‘crc’. The six developing or decreasing themes in the bottom left quadrant, which include ‘intrahepatic cholangiocarcinoma’, ‘myc’, ‘fibrosis’, ‘oral lichen planus’, ‘classical hodgkin lymphoma’, and ‘prognostic signature’, are marked by low centrality and density, suggesting poorly developed and marginal themes.

## 4 Discussion

Based on available data, this study summarizes research collaborations, current hotspots, new developments, and research trends in TSA-based mIHC/IF throughout the world. Overall, 672 English-language articles and reviews were obtained in this bibliometrics analysis. The first article applied TSA-based mIHC/IF was published in 2007 (23), but it wasn’t until 2016 that TSA-based mIHC/IF took off, and the number of insightful publications and the range of applied disciplines increased exponentially. This may be partly due to the marketing of commercial kits and the use of clinical FFPE samples (3, 25, 26). The USA and the University of Texas MD Anderson Cancer Center are the most productive and influential parties, contributing the highest number of publications and citations. It is worth mentioning that since 2017, China has begun to publish articles applied this method, and in the following years, the number of publications and citations has increased rapidly. In 2022, the total number of publications and citations reached 184 and 1722, respectively, placing second. Moreover, Fudan University (Rank 2), Capital Medical University (Rank 3), and Sun Yat-sen University (Rank 9) are in the top 10 institutions ranking list. It indicates that a growing number of Chinese institutions have created an experimental platform of TSA-based mIHC/IF to address diverse scientific challenges and generate high-quality papers.

The cutting-edge and trending concerns in TSA-based mIHC/IF are elucidated by co-citation and keyword analysis. The bulk of research was conducted in the fields of neuroscience, oncology, and immunology. As early as 2008, Ildiko Racz et al. applied fluorescein isothiocyanate TSA method to double staining for CB2 receptors and for microglia or for astrocytes, aiming to study the critical role of the CB2 cannabinoid receptor in the regulation of central immune responses during neuropathic pain (27). The TSA method was initially used in neuroscience research for the simultaneous labeling of multiple targets (28–36). TSA-based mIHC/IF has since gained popularity and interest among oncology researchers and is progressively becoming an essential tool for characterizing the TME of various cancers, in particular colorectal cancer (37, 38), oral squamous cell carcinoma (39, 40), melanoma (16, 41, 42) and cervical cancer (43–45), as indicated by the references and keyword analyses. Many different panels have been established for clinical research, in which the importance of tumor immune infiltrate densities, cell phenotypes, spatial localization, tertiary lymphoid structures, and the identification of targetable biomarkers for prognostic patterns, such as PD-L1, have been analyzed to study the effect of immunotherapy and potentially help to better match patients to appropriate treatment regimens (15, 46, 47). Since 2021, TSA-based mIHC/IF has been dramatically integrated with the omics technologies, such as single-cell sequencing (48–51) and mass spectrometry-based proteomics technologies (52, 53), in the preclinical research and clinical application to deepen the understanding of tumorigenesis, tumor progression, and responses to immunotherapy. Spatial proteomics, spatial transcriptomics, and spatial metabolomics have all gained popularity as a result of the ongoing advancements in spatial omics technologies. In addition to providing high-resolution and detailed analysis for single-cell omics approaches, spatial omics adds a spatial component that is essential for comprehending the intricate architecture and functional linkages found in biological systems (54). For spatial proteomics, the technology can be categorized based on the antibody labeling method (55). Firstly, one of the most popular techniques for moderate-to-low throughput *in situ* protein detection is the use of fluorescently labeled antibodies to detect multi-proteins This method allows for the visualization of multiple proteins within a single tissue section, such as TSA-based mIHC/IF, tissue-based cyclic immunofluorescence (t-CyCIF), iterative bleaching extends multiplexity (IBEX), iterative indirect immunofluorescence imaging (4i) (3, 56–58). Additionally, cyclic staining, antibody modification, and specialized detection equipment are needed when using DNA Barcode-labeled antibodies to detect multi-proteins like the CODEX (Co-Detection by indexing) platform (59). This approach offers higher multiplexing capabilities but comes with higher costs due to the need for advanced instrumentation and reagents. For high-throughput protein omics, such as Imaging Mass Cytometry (IMC) which used metal-labeled antibodies to overcomes the limitations of fluorescent detection channels (60). However, IMC also necessitates specific antibodies and tools, which raises capital and operating costs. While these methodologies are powerful tools for spatial proteomics, the type of sample, the goals of the study, practicality, and cost-effectiveness should all be considered when selecting a technology.

With the significant advantage of detecting multiple targets simultaneously, TSA-based mIHC/IF provides powerful tools and methods against the studies of immunopathology and neuroinflammation of SARS-CoV-2 and COVID-19 (61–66). Using TSA-based mIHC/IF, the researchers have highlighted novel immunopathological mechanisms and pathological changes in the pancreas (64) and the central nervous system (61, 66). These findings will broaden the investigation of therapeutic targeting of aberrant cellular immune responses and facilitate clinical treatment in COVID-19.

Over the past few decades, TSA-based mIHC/IF has evolved fast. Researchers have completed a variety of processes, including slide preparation, antibody optimization, the design, validation, and standardization of a marker panel, and manual and automated staining protocol (8, 24, 41, 67–75). As pathology has advanced into the digital age, image acquisition has broadened from capturing small fields of view to scanning entire slides. This gets significantly more difficult when using multiplex fluorescent staining imaging. Spectral unmixing techniques address this challenge by extracting the specific spectral profiles of individual fluorophores and using unmixing algorithms to resolve overlapping fluorescence signals (76). Commercial platforms such as the PhenoImager HT system from Akoya Biosciences employ these advanced algorithms to achieve high-precision signal separation. Additionally, the use of custom-designed narrow-band filter sets can effectively mitigate the problem of spectral overlap (24). With carefully selecting and combining filters that allow only specific wavelengths to pass, it is possible to minimize cross-talk between different fluorescence channels, thereby enhancing the clarity and accuracy of the images. Currently, there are multiple commercial software, such as HALO (Indica Labs), inForm (Akoya Biosciences), and Visopharm TissueAlign (Visiopharm), as well as some open-source platforms like Qupath (77), histoCAT (78), CellProfiler (79), which can be widely used for the analysis of multiplex fluorescence staining images. Cell Segmentation, Tissue classification, Phenotype Characterization, and Positive Cell Rate/Expression Intensity Analysis are all common steps in the workflow for these investigations (80). Artificial intelligence (AI) technologies are increasingly being integrated into the analysis pipeline to assist with nuclear segmentation, significantly improving the accuracy of cell quantification (81). Moreover, the analysis of cell-to-cell distances and intercellular interactions has gained prominence, offering a better understanding of cellular microenvironments (82). To further enhance the comprehensiveness of tissue microenvironment investigation, a growing number of analytical algorithms have been developed using the R programming language (83). In order to provide a more comprehensive understanding of biological processes, these algorithms enable more intricate and nuanced interpretations of spatial linkages and molecular interactions inside tissues.

The TSA-based mIHC technique offers notable advantages in application, including the capacity for analysis in FFPE (Formalin-Fixed Paraffin-Embedded) specimens, reduced reagent expenses relative to alternative multi-omics profiling methodologies. This technique supports implementation through either automated and manual detection processes. Due to its protocol bearing a strong resemblance to traditional immunohistochemical staining methods, the technology demands minimal specialized technical skill. However, considering epitope damage, signal loss and tissue integrity, each staining step still need intensive work to optimize. Specialized training, standard operating procedure and extensive cooperation will facilitate the dissemination and application of this technology (2, 3, 8, 84).

Using the WOSCC database and three bibliometric approaches, this study has compiled detailed information on the application of TSA-based mIHC/IF between 2007 and 2023, including publication volume and growth patterns, journals, countries/regions, institutions, authors, references, and keywords. However, several inevitable limitations of this study should be considered. To begin, searching and downloading literature from the WOSCC database may result in the omission of relevant studies from other databases. Secondly, the screening was confined to the English language. Therefore, non-English publications may receive insufficient attention. Nonetheless, this study still covers the majority of publications on TSA-based mIHC/IF and provides valuable insights into current research hotspots, evolutionary processes and trends in the field.

In conclusion, this is the first bibliometric analysis of the application of TSA-based mIHC/IF, exploring the features of the publications from 2007 to 2023 and revealing the current research priorities and future directions. With the promotion of transdisciplinary and technical progress, TSA-based mIHC/IF and its associated imaging systems and analysis software will continue to improve, broadening the scope of application and improving the depth and accuracy of analysis. This method has a growing impact and emerging applications in basic and translational research as well as in clinical medicine, based on its important ability to characterize the diversity of the entire tissue for a better understanding of pathological or physiological behavior, and with the ultimate goal of implementation in the clinical diagnosis and treatment of multiple diseases.

## Data Availability

The original contributions presented in the study are included in the article/supplementary material. Further inquiries can be directed to the corresponding author.
